# The role of ONSD in the assessment of headache associated with Chiari malformation type 1

**DOI:** 10.3389/fneur.2023.1127279

**Published:** 2023-02-07

**Authors:** Mehmet Kürşat Karadag, Mehmet Emin Akyuz, Mehmet Hakan Sahin

**Affiliations:** Neurosurgery Department, School of Medicine, Atatürk University, Erzurum, Türkiye

**Keywords:** Chiari malformation type 1, optic nerve sheath diameter, headache, intracranial pressure, nuchal pain

## Abstract

**Background:**

Cough associated headache is the most common symptom in Chiari malformation type 1 (CM1). However, its pathophysiology and treatment are not clear. The aim of this study was to investigate the relationship between optic nerve sheath diameter (ONSD), an indicator of intracranial pressure, and headache and to investigate its predictive value on postoperative outcome.

**Methods:**

In symptomatic CM1 patients, craniovertebral junction morphometric measurements and ONSD measurements were made from preoperative MR images, and headache intensities and characteristics were evaluated. After different surgical procedures, the clinical characteristics of the patients were evaluated according to the Chicago Chiari Outcome Scale, the change in headache intensity was assessed and the relationship with ONSD was evaluated.

**Results:**

Preoperative headache intensity was significantly correlated with ONSD measurement (*p* < 0.01). Modified clivoaxial angle and ONSD were independent predictors of postoperative clinical outcome (*p* < 0.01). The procedure that achieved the greatest surgical enlargement of the foramen Magnum stenosis provided the best clinical outcome. Postoperative reduction in headache intensity and ONS diameter were correlated (*p* < 0.01).

**Conclusion:**

The fact that ONSD is found to be wide in the preoperative period in CM1 patients indicates that the intracranial pressure is permanently high. This diameter increase is correlated with headache and is a valuable guide in the selection of the appropriate treatment method.

## Introduction

Chiari malformation type 1 (CM 1) is a pathology characterized by herniation of the cerebellar tonsils from the foramen magnum (1). This herniation puts pressure on the spinal cord and brainstem, and causes a blockage of actual or functional cerebrospinal fluid (CSF) (2). With the increase in the number of imaging studies, more patients are diagnosed with CM1, however, only 1% of these patients are symptomatic (3).

Cerebellar tonsil ectopia may interfere with the normal cranio-spinal movement of the CSF, causing increased headache in these individuals with laughing, coughing or tilting the head, or the valsalva maneuver. Also, tonsillar compression on the brain stem and upper cervical spinal cord can cause varying degrees of somatosensory disorders and subtle motor defects, as well as bulbar and lower cranial nerve disorders (4). Various theories have been proposed for CM1-related headache, some of which are; The Oldfield's theory describes pulsatile caudal pressure that occurs with mechanical contact on the spinal cord (5), William's theory describes that increased abdominal pressure occurs as a result of a craniocaudal pressure gradient that cannot diffuse into the subarachnoid space. In Williams' theory, cerebrospinal fluid flow is blocked in the subarachnoid space at the foramen magnum, and a longer-lasting venous pressure wave associated with coughing and straining affects the cord, first to compress it from the outside, then to expand it from the inside. This causes a transient increase in intracranial pressure and headache with cascading stimulation of the trigeminal afferents of the dura (6).

Optic nerve sheath diameter (ONSD) measurement has been studied many times in the literature as an indirect indicator of increased intracranial pressure (7, 8). When all the English literature was reviewed, we could not find a study on the measurement of optic nerve sheath diameter in the evaluation of clinical outcomes of Chiari malformation. However, we think that this measurement will be very important in clinical evaluation. The aim of this study is to investigate whether there is a permanent change in intracranial pressure in CM1-related headache, to examine the effect on headache intensity and duration if there is, and to investigate whether there is a change after different surgical procedures.

## Methods

### Patients

In this study, 195 patients who were operated for CM1 between 2015 and 2020 years in our clinic were evaluated retrospectively. The study was started after obtaining the approval of the local ethics committee of Atatürk University, Faculty of Medicine (B.30.2.ATA.0.01.00/661). The inclusion criteria were as follows; being older than 18 years, radiologically proven cerebellar tonsil herniation more than 5 mm from the foramen magnum. The exclusion criteria were: presence of secondary causes for headache (intracranial idiopathic hypertension, etc.), presence of intracranial lesion (subdural hematoma, space occupying lesion, etc.), other anomalies of the craniovertebral junction (atlantoaxial dislocation, basilar invagination, etc.) and prominent hydrocephalus (Evans index >3).

### Surgical technique

The patient was placed on the surgical table in the prone position with the head fixed. intervention was performed with a classical suboccipital incision and laminectomy was performed with suboccipital craniectomy and removal of the C1 posterior arch (PFD group). In some patients, the dura was opened in a U shape and dural augmentation was performed with a dural graft (PFDwD group). In some patients, the cerebellar tonsils were resected until the foramen Magendie was seen and CSF flow was achieved (CTR group). All surgical procedures were performed by an experienced team under the supervision of a senior author (HHK). The surgical procedure was decided according to the herniation length of the cerebellar tonsil and the stenosis of the occipitocervical junction as a result of morphometric measurements.

### Preoperative radiological measurements

**Figure 1:**
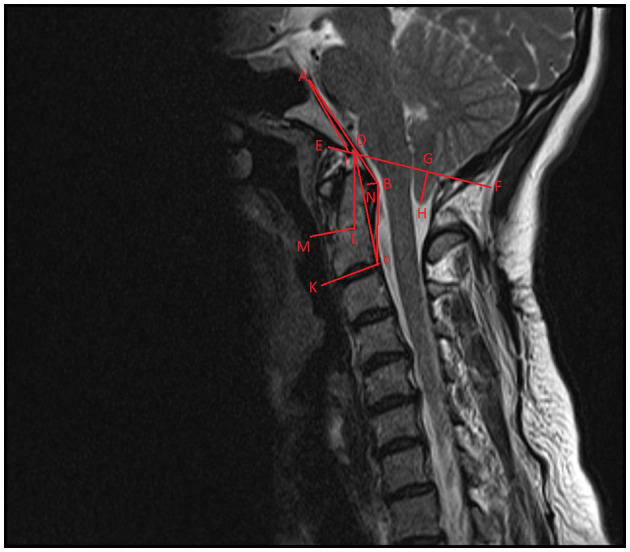
Schematization of craniovertebral morphometric measurements on sagittal T-2-weighted imaging. ABC, modified clivoaxial angle; ADC, clivoaxial angle; GH, herniated cerebellar tonsil length; AD, clivus length; AIE, clivus angle; DCK, odontoid retroversion; DLM, odontoid retroflexion; DF, McRae line.

Preoperative radiological evaluations were made with MRI taken while the neck was in the neutral position. The morphometric measured values are schematized in Figure 1. Optic nerve sheath diameter was assessed by measuring the thickest part of the optic nerve sheath 3 mm posterior to the globe on T1-weighted axial MRI sections. It was measured for both eyes separately and the average was recorded as the final value. Preoperative MRI and postoperative 6th month MRI images were recorded.

### Outcome measures and assessment of headache

Postoperative clinical evaluation was done with Chicago Chiari Outcome Scale (CCOS), includes pain, non-pain symptoms, functionality, and complications), and then categorized as improved (score 13–16), unchanged (score 9–12), or worse (4–8) based on the outcome score (9). This evaluation was made at 24 months postoperatively. According to the improvement in postoperative CCOS scores, patients will be grouped according to their preoperative ONSD values (increased intracranial pressure) using the ROC curve.

Preoperative headache was obtained by retrospective review of patient files. Characteristics such as the type of headache (headache increasing with cough), localization, severity, frequency, and the number of days of the month were determined. Postoperative headache assessment was made 1 year or more after surgery, by completing a questionnaire during a telephone interview or hospital visit. In the postoperative evaluation, it was asked to evaluate the severity of pain numerically (0 = no pain, 10 = the most severe pain), and also the localization of the pain, its frequency, and whether medication was taken, pain had changed after the surgical procedure (worsened, not changed, improved). Headache semiology was decided according to the International Classification of Headache Disorders 3rd edition (ICHD-3) (10). Typical headache attributable to CIM (according to the ICHD-3 diagnostic criteria, according to section) was described as a (sub-)occipital headache lasting seconds to a maximum of 5 min, triggered by coughing, laughing, or other Valsalva-type maneuvers.

### Statistical analysis

Statistical evaluation was performed with the statistical program (SPSS Statistics, version 22, IBM, Armonk, NY). Wilcoxon's test was used for comparison of pre- and postoperative data, and the Mann–Whitney U test, or Chi-square test were used for comparisons between groups. Dichotomous variables were compared pre- and postoperatively using the McNemar test. Bivariate analysis (Pearson's and Spearman's correlation analysis) and multiple linear regression model were used to analyze the correlation between the preoperative ONSD and other MRI parameters. The correlation between ONSD and postoperative CCOS scores and headache intensity was examined by bivariate analysis, regardless of the surgical method. The receiver operating characteristic curve was used to test the accuracy of preoperative ONSD measurements in predicting CCOS score and identifying optimal thresholds of ONSD. For the same ONSD measurements group, the difference of CCOS score and headache intensity of 3 surgical methods was analyzed by one-way analysis of variance.

## Results

A total of 195 patients who were operated on for CM1 were included in this study, 100 of them were female and 95 were male. Mean age of patients was 42 ± 4 years, mean follow-up time was 44 ± 8 months. The mean cerebellar tonsil length that herniated from the foramen magnum was 10.8 + 4.2 mm.

The mean CCOS score of the patients was 12.74 ± 3.1 in general, among them, the symptoms of 117 (60%) patients improved, 68 patients (34.8%) were the unchanged, and 10 patients (5.1%) worsened. In terms of surgical technique, improvement (≥13) from the CCOS score was seen as 50.0% in the PFD group (28 of 56), 59.2% in the PFDwD group (32 of 54), and 78.8% in the CTR group (67 of 85), and mean CCOS scores were 12.24 ± 2.3, 13.1 ± 1.2, and 14.1 ± 1.8, respectively. There was a statistically significant difference in terms of outcome scores between these (*p* < 0.05). Complications were observed in 28 of these cases, 14 with wound site problems without CSF leak, 10 with CSF leak, 1 deep vein thrombosis, 1 pulmonary embolism, and 2 deaths due to cerebellar infarction (PICA injury).

According to the results of bivariate analysis, ONSD was statistically significantly correlated with; MCAA, clivus length, clivus angle, odontoid retroflexion, odontoid retroversion, pB-C2 distance, tonsillar herniation, and cranial basal angle (*p* < 0.05) (Table 1). Multiple linear regression analysis showed that MCAA (-), clivus angle (+), pB-C2 distance (–), cranial basal angle (–), and odontoid retroversion (–) correlated independently (*p* < 0.05) (Table 2).

**Table 1:** Correlation of ONSD with other parameters by bivariate analysis, *p* < 0.05 is statistically significance.

**Variable**	**Value**	**ρ/r**	***p*-value**
ONSD	5.42 ± 0.27	1.00	NA
MCAA°	134.33 ± 10.27	−0.37	<0.01
pB-C2 distance (mm)	8.63 ± 1.15	−0.47	<0.01
Cranial base angle (°)	122.42 ± 7.41	−0.78	<0.01
Clivus length (mm)	43.03 ± 2.46	0.39	<0.01
Clivus angle (°)	45.24 ± 8.32	0.46	1.37
Tonsillar herniation (mm)	12.31 ± 5.20	−0.15	0.03
Odontoid retroversion (°)	93.08 ± 5.63	−0.32	<0.01
Odontoid retroflexion (°)	108.52 ± 8.10	−0.29	<0.01
McRae line (mm)	34.48 ± 3.70	−0.05	0.38
Illness duration (month)	44.46 ± 79.85	0.07	0.37
Operation (PFD/PFDwD/CTR)	64/60/91	0.08	0.37
BMI (kg/m^2^)	28.20 ± 4.07	0.01	0.79
Age (years)	44.77 ± 10.37	0.07	0.29
Sex (female/male)	100/95	0.02	0.72

NA, not applicable; ρ/r, correlation coefficient.

**Table 2:** Correlation of ONSD with other parameters by multiple linear regression model, *p* < 0.05 is statistically significance.

**Variable**	**B**	**Beta**	***P***	**95% CI**	
				**Lower**	**Upper**
Constant	224.52	NA	<0.01	202.54	236.41
MCAA(°)	−1.72	−0.31	<0.01	−2.44	−1.39
pB-C2 distance (mm)	−1.82	−0.33	<0.01	−2.42	−1.41
Cranial base angle (°)	−0.41	−0.51	<0.01	−0.52	−0.44
Clivus length (mm)	0.21	0.05	0.26	**–**0.04	0.30
Tonsillar herniation (mm)	0.03	0.02	0.58	**–**0.11	0.17
Clivus angle (°)	0.27	0.35	<0.01	0.10	0.48
Odontoid retroversion (°)	−0.18	0.06	0.02	−0.49	−0.02
Odontoid retroflexion (°)	**–**0.07	**–**0.04	0.16	**–**0.31	0.04

B, partial regression coefficient; Beta, standardized partial regression coefficient, NA, not applicable; CI, confidence interval.

When evaluated in terms of postoperative CCOS score, preoperative ONSD, preoperative MCAA, clivus angle, pB-C2 distance, cranial base angle, and odontoid retroflexion were statistically significantly correlated according to the bivariate analysis (*p* < 0.05) (Table 3), ONSD and MCAA were independently correlated according to multiple linear regression analysis under the dummy variables of surgery type (*p* < 0.05) (Table 4).

**Table 3:** The bivariate analysis of ONSD and other craniovertebral junction parameters with CCOS.

**Variable**	**Value**	**r**	***P***
CCOS	12.64 ± 2.1	1	NA
ONSD (mm)	5.42 ± 0.27	0.42	<0.01
MCAA (°)	134.33 ± 10.27	0.52	<0.01
Clivus angle (°)	45.24 ± 8.32	0.17	<0.01
pB-C2 distance (mm)	8.63 ± 1.15	−0.27	<0.01
Cranial base angle (°)	122.42 ± 7.41	−0.29	<0.01
Clivus length (mm)	43.03 ± 2.46	0.27	<0.01
Tonsillar herniation (mm)	12.31 ± 5.20	**–**0.14	0.12
Odontoid retroversion (°)	93.08 ± 5.63	**–**0.89	0.26
Odontoid retroflexion (°)	108.52 ± 8.10	−0.16	0.03

**Table 4:** The multiple linear regression modal of ONSD and other craniovertebral junction parameters with CCOS (operations used as dumb variable).

**Variable**	**B**	**Beta**	***P***	**95% CI**	
				**Lower**	**Upper**
ONSD	0.06	0.81	<0.01	0.03	0.94
MCAA (°)	0.07	0.83	<0.01	0.04	0.95
Clivus angle (°)	**–**0.02	**–**0.04	0.47	**–**0.07	0.01
pB-C2 distance (mm)	0.04	0.03	0.22	**–**0.04	0.19
Cranial base angle (°)	0.02	0.21	0.26	**–**0.01	0.03
Clivus length (mm)	0.03	0.17	0.15	**–**0.01	0.08
Odontoid retroflexion (°)	**–**0.01	**–**0.06	0.47	**–**0.04	0.02
Operation 1	0.75	0.02	2.08	0.00	0.27
Operation 2	1.00	0.04	2.65	0.00	0.65

B, partial regression coefficient; Beta, standardized partial regression coefficient; CI, confidence interval.

**Figure 2:**
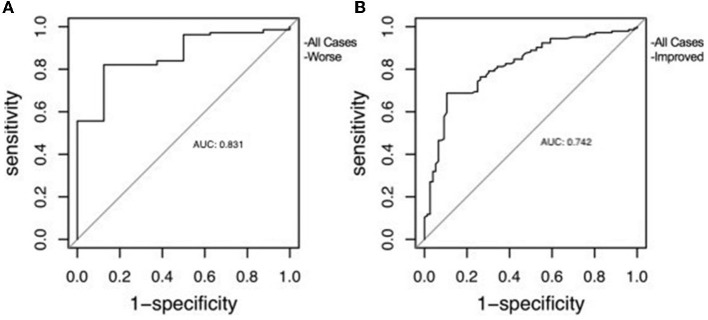
The receiver operating characteristic curve of ONSD used to predict clinical outcome. **(A)** Correlation with clinical worsening. **(B)** Correlation with clinical improvement.

Performed to confirm the predictive efficacy of ONSD over CCOS with ROC curve, the results were quite good at predicting CCOS. The results were as follows, for prediction worse: AUC (area under curve) = 0.831, *p* < 0.01, for prediction improved: AUC = 0.742, *p* < 0.01. Also according to Youden's index, the optimal threshold value of ONSD was 5.54 and 4.72 mm, respectively (Figure 2).

The patients were divided into three groups according to the optimal threshold value of ONSD to assess preoperative intracranial pressure value; A = severe (ONSD ≥ 5.54) in 39 cases, B = moderate (4.72 ≤ ONSD <5.54) in 78 cases and C = mild (ONSD <4.72) in 78 cases.

The mean CCOS scores of the groups were 11.69 ± 1.2, 12.85 ± 1.4, 13.61 ± 1.7, respectively, and there was a statistically significant difference between the groups. Among these groups, Group C showed the most improvement and there was a statistically significant difference in this respect (Table 5).

**Table 5:** Mean CCOS scores and percentages of change of patients grouped by ONSD scores.

	**Group A**	**Group B**	**Group C**	***p*-value**
Mean CCOS score	11.69 ± 1.2	12.85 ± 1.4	13.61 ± 1.7	<0.01
Improved (*n*/%)	12/30.7%	34/43.5%	70/89.7%	<0.01
Unchanged (*n*/%)	19/48.7%	42/53.8%	8/10.3%	<0.01
Worsed (*n*/%)	8/20.5%	2/2.5%	0/0	<0.01

Headache was the predominant symptom in 120 of 195 patients. The baseline characters of headache are shown in Table 6. Headache was predominantly occipital or nuchal (79.9%) and bilateral (80.0%), and headache quality was mostly pressing (68.3%). The most common accompanying symptom was nausea with 43.3%. Average minimum headache intensity was 4.5 ± 1.4 on the NRS and mean maximum intensity was 7.9 ± 1.5. The number of days the headache persisted in a month was 21.4 ± 9.1 and the number of days on which medication was used for headache was 10.1 ± 7.9. With regard to semiology according to ICHD-3, typical cough headache was seen in 62 patients (51.6%). Headache was present with other (“atypical”) semiology in 58 patients (48.3%). Preoperative headache intensity was found to be highest in the severe ONSD group. The preoperative mean headache intensity was 7.1 ± 1.2 in the severe ONSD group (*p* < 0.05).

**Table 6:** Baseline headache characteristics (*n* = 120).

**Headache characteristics (*****n*** = **41)** ***n*** **(%)**	
**Exacerbation during**	
Physical activity	95 (79.1)
Rising up	62 (51.6)
Cough/laughing/Valsalva maneuver	78 (65.0)
Lying down	10 (8.3)
**Duration**	
Seconds to minutes	72 (60.0)
Minutes to hours	12 (10.0)
Hours to days	36 (30.0)
**Side preference**	
Bilateral	96 (80.0)
Left	13 (10.8)
Right	8 (6.6)
**Localization**	
Nuchal	52 (43.3)
Occipital	43 (36.6)
Frontal	10 (8.3)
Temporal or parietal	8 (6.6)
Holocephalic	7 (5.8)
**Pain quality**	
Pressing	82 (68.3)
Pulsating	10 (8.3)
Stabbing	10 (8.3)
Pulling	15 (15.0)
**Accompanying symptoms**	
Nausea	52 (43.3)
Vomiting	29 (24.1)
Photophobia	20 (16.6)
Phonophobia	20 (16.6)
Migraine aura	18 (15.0)
Hypacusis	9 (7.5)
Visual impairment	28 (23.3)
**Headache semiology (according to ICHD-3)**	
“Typical” cough headache	62 (51.6)
“Atypical” headache semiology	58 (48.3)

After surgery, subjective headache relief was noted in 104 patients (86.6%), while headache remained unchanged in 8 cases (6.6%), and worsened in 8 patients (6.6%). When the correlation between postoperative ONSD and decreased headache intensity was examined, it was seen that the lower the ONSD value, the lower the headache intensity. The relationship between surgical technique and headache intensity outcome is shown in Table 7.

**Table 7:** Variation of mean ONSD, headache intensity scores and CCOS scores between groups after 3 different surgical approaches.

	**Surgical technique**			***p*-value**
	**PFD**	**PFDwD**	**CTR**	
Mean ONSD (mm)	6.2 ± 1.1	5.4 ± 1.3	4.4 ± 1.1	<0.01
Headache intensity (NRS)	6.7 ± 1.2	5.8 ± 1.3	4.9 ± 1.2	<0.01
CCOS score	12.24 ± 2.3	13.1 ± 1.2	14.1 ± 1.8	<0.01

## Discussion

The aim of this study is to reveal the unexplained and missing part in the pathophysiology of headache associated with Chiari malformation. The most important inference that can be drawn from the study is that CSF blocks that occur as attacks in CM1 patients cause permanent increases in intracranial pressure. In these patients, ONSD, an indirect marker of intracranial pressure, was found to be increased and directly associated with headache intensity.

CM1 is a hindbrain anomaly and is a mostly congenital pathology characterized by varying degrees of herniation of the cerebellar tonsils from the foramen magnum (11). Clinical signs and symptoms can vary widely depending on the crowding and blockage in the foromen magnum. Tonsillar herniation and various bone and soft tissue defects in the craniovertebral junction cause compression on the spinal cord and brain stem, resulting in clinical manifestations. This crowding at the craniovertebral junction causes functional or actual blockade of the CSF at the level of the foroman Magnum (12).

Many studies show that headache is the most common complaint in CM1 patients (13). Milhorat et al., in their study by observing 364 patients, stated that 86% of these patients had headache and most commonly localized pain in the occipital and nuchal region, exacerbated by valsalva-like maneuvers, and in the form of crushing and pressure (14). Similarly, Thunstedt et al. reported that 63% of CM1 patients had headache, and 80% of this headache was localized in the occipital and nuchal regions (15). In our study, we found that there was a 61% headache and the localization was in a similar region. We also found that the most common accompanying symptom was nausea, as in the Thunstedt's study, and 51% of those with headache had typical cough-related headaches.

Predictive factors for clinical improvement after surgery in symptomatic CM1 patients have been investigated in many studies and continue to be investigated. Morphometric measurements of the craniovertebral junction are used to show the compression of the foromen magnum (16). However, many studies are mostly based on examining bone pathologies. However, the soft tissues in the retroodontidal space cause ventral compression depending on the anatomical position and angulation of the odontoid process (17). The modified clivoaxial angle, which assesses both the relationship of the odontoid process to the brainstem and the pressure created by the soft tissues (transverse ligament and tectorial membrane) in the retroodontoidal space, is very successful in predicting postoperative clinical improvement (18). In our study, we found that MCAA was very successful in predicting the postoperative CCOS score.

It is generally accepted that cough-related headache in CM1 patients is caused by impaired CSF flow between the head and spinal cord due to compression of the foramen Magnum (19). In their study, Williams et al. showed that CSF pressure in the spinal cord increased with increasing intra-thoracic and intra-abdominal pressure at the onset of cough, and this increase in pressure caused CSF flow toward the cranium, and when the cough ended in healthy individuals, the cranial CSF returned to the spinal canal, but this return did not occur in CM1 patients due to the crowding in the foramen magnum (6). However, no study to date has investigated whether this transient CSF flow disturbance causes a change in long-term intracranial pressure. The typical cough-related headache in Chiari patients is explained by the above-mentioned mechanism, but the physiopathology of atypical headache remains unclear. ONSD has been used in many diseases and clinical studies as a very important indicator of increased intracranial pressure; such as shunt dysfunction, hydrocephalus, posttraumatic intracranial pressure monitoring (7, 20–22). When the preoperative MR images of the patients were examined, we found that the ONS diameters were significantly higher than the accepted values in normal healthy individuals and correlated with headache intensity (NRS). When the English literature is reviewed, such a finding has not been studied before, and we think that it will be very important in understanding the pathophysiology of CM1-related headache.

In their study, Fric et al. showed that pulsatile intracranial pressure in CM1 patients was high, just like in patients with idiopathic intracranial hypertension (23). In their study, Fagan et al. found that the symptoms, especially headache and balance disorders, did not improve after posterior fossa decompression, but the symptoms regressed after CSF diversion and named this condition Chiari pseudotumor cerebri syndrome (24). In Fric's study, they mentioned that pulsatile intracranial pressure is a better indicator of intracranial compliance than static intracranial pressure, they found that static intracranial pressure was not as high in CM1 patients as in IIH patients in their cohort study. However, including their own cohort studies, lumbar puncture to measure intracranial pressure is very risky in CM1 patients because of tonsillar herniation in the preoperative period, and measuring pressure *via* the intraventricular route in the absence of ventriculomegaly is also very risky. The measurement of ONSD is a non-invasive method and a good indicator of intracranial pressure, the results of the study show that not only pulsatile pressure but also static pressure is high in CM1 patients.

In CM1 patients, only posterior fossa decompression does not always provide the desired level of clinical improvement (25). Especially in patients with ventral compression, it is necessary to extend the posterior fossa decompression as far as the CTR to resolve the foramen Magnum crowding and to allow CSF flow to normal limits. When the effect of surgical procedures on clinical outcome was analyzed, it was observed that both CCOS was higher and headache intensity was less in patients who underwent CTR. Although clinical output improves more after CTR, the duration of surgery is prolonged and the risk of postoperative complications increases (26). Giammattia et al. described an 86.9% improvement in headache after surgery in their study (27). In our study, the improvement in symptoms was ~60% and this rate is the average of the results of all three surgical techniques. CTR with visualization of the foramen Magendie and observation of CSF flow showed much better results. As stated in the literature, we think that tonsil resection until the foramen Magendie is seen would have a positive effect on all results (28). In the choice of surgical procedure, knowing the preoperative intracranial pressure level and foramen Magnum stenosis is very valuable in deciding which method to choose. ONSD and MCCA seem to be useful guides in this choice.

### Limitations

The most important limitations of this study are the relatively small number of patients, retrospective observation, and the fact that intracranial pressure in CM1 patients was not routinely measured in the preoperative and postoperative periods, which necessitated the assessment of pressure status and variability by indirect methods. A prospective study model with a larger number of patients in which intracranial pressure measurements can be performed may be more useful. An important shortcoming of this study is that syringomyelia was not evaluated. In fact, the reason for not evaluating it was the concern that if too many parameters were included, the article would lose its integrity of meaning. As a continuation of this study, the relationship between ONSD and syringomyelia will be examined.

## Conclusion

Chiari malformation type 1 is a developmental disorder involving not only the displacement of the cerebellar tonsils but also the bone and soft tissue of the craniovertebral junction. This crowding in the foramen magnum disrupts the normal circulatory physiology of the CSF, resulting in clinical symptoms such as headache. Increased intracranial pressure in CM1-related headache has been shown by ONSD measurements. We think that routine evaluation of ONS diameter in the preoperative period can be very useful in deciding the surgical method to be chosen and it is also useful in determining whether additional intervention (CSF diversion, etc.) is required in postoperative follow-up.

## Data availability statement

The raw data supporting the conclusions of this article will be made available by the authors, without undue reservation.

## Ethics statement

The studies involving human participants were reviewed and approved by the Ethics Committee of Ataturk University, School of Medicine (B.30.2.ATA.0.01.00/661). Written informed consent from the patients/participants or patients/participants' legal guardian/next of kin was not required to participate in this study in accordance with the national legislation and the institutional requirements.

## Author contributions

Material preparation, data collection, and analysis were performed by MA and MK. The first draft of the manuscript was written by MA. Evaluation and supervision of the article was done by MK and MS. All authors commented on previous versions of the manuscript, contributed to the study conception and design, and read and approved the final manuscript.
